# Hepatitis B prevalence, risk factors, infection awareness and disease knowledge among inmates: a cross-sectional study in Switzerland’s largest pre-trial prison

**DOI:** 10.7189/jogh-08-020407

**Published:** 2018-12

**Authors:** Laurent Gétaz, Alejandra Casillas, Claire-Anne Siegrist, François Chappuis, Giuseppe Togni, Nguyen-Toan Tran, Stéphanie Baggio, Francesco Negro, Jean-Michel Gaspoz, Hans Wolff

**Affiliations:** 1 Division of Prison Health, Geneva University Hospitals and University of Geneva, Geneva, Switzerland; 2 Division of Tropical and Humanitarian Medicine, Geneva University Hospitals and University of Geneva, Geneva, Switzerland; 3 Department of Pathology and Immunology, University of Geneva, Geneva, Switzerland; 4 World Health Organization Collaborating Centre for Vaccine Immunology, University of Geneva, Geneva, Switzerland; 5 Unilabs, Coppet, Switzerland; 6 Australian Centre for Public and Population Health Research, Faculty of Health, University of Technology, Sydney, Australia; 7 Division of Gastroenterology and Hepatology and Division of Clinical Pathology, University Hospital, Geneva, Switzerland; 8 Department of Primary Care, Community Medicine, and Emergencies, Geneva University Hospitals and University of Geneva, Geneva, Switzerland

## Abstract

**Background:**

Hepatitis B virus (HBV) is a major health concern in prison, but data are scarce in European prisons. This study aims to measure the prevalence of HBV infection, risk factors, awareness about infection, and HBV knowledge among inmates in Switzerland’s largest pre-trial prison.

**Methods:**

Serological blood tests (HBsAg, anti-HBs, and anti-HBc) and a standardized socio-demographic and sexual health survey were offered to consenting prisoners in 2009 and 2011. HBV knowledge was assessed using a standardized questionnaire among participants recruited in 2009.

**Findings:**

A total of 273 male participants were included in the study (116 participants answered the HBV knowledge survey), with 38.1% originating from Eastern Europe, 28.2% from sub-Saharan Africa, 14.3% from North Africa, and 9.5% from Latin America. The prevalence of anti-HBc (resolved/chronic infection) was 38.2% and the prevalence of HBsAg (chronic infection) was 5.9%; 14% of participants had vaccine-acquired immunity (anti-HBs positive/anti-HBc negative). We estimated that 15.5% of people living in Geneva having chronic infection go through the Geneva’s prison. Region of origin was significantly associated with chronic/resolved HBV infection (*P* < 0.001): 72.2% of participants from sub-Saharan African, 34.6% from Eastern Europe and 13.2% from other regions. In terms of chronic infection, 15.6% of participants from sub-Saharan Africa were positive for HBsAg, vs 2% of those from other regions (*P* < 0.001). In stratified analyses, region of origin remained significantly associated with HBV infection. Among those with chronic infection, only 12.5% were aware of their status. A minority of inmates knew how HBV could be transmitted.

**Conclusions:**

The primary factor associated with HBV infection in this study was the geographical region of origin of participants. Given the high HBV prevalence found in this prison population, a targeted testing and vaccination approach based on prisoners’ region of origin would be a cost-effective strategy when resources are limited. Additionally, identification of at-risk people should not rely on sensitive questions nor self-reported history of HBV. An inclusive approach to global health needs to incorporate prison population, as incarcerated people have a disproportionate burden of HBV infection and because an important proportion of hard-to-reach chronic HBV infected people go through the incarceration system.

Hepatitis B virus (HBV) infection is a significant public health problem in prison settings with a global incidence estimate of 0.8%-3.8% per year [1-3]. Studies in the United States show that up to 3.7% of prisoners suffer from chronic hepatitis B. The proportion is higher in European prisons (4.4% in Denmark, 6.5% in Belgium) [4]. The prevalence of anti-HBc antibodies (indicating infection at some point in time) in prisoners is 20%-25% in the United States with a wider range in Europe (9% in Ireland, 58% in Greece) [4,5].

There is a higher risk of HBV transmission in prison settings due to the lack of knowledge about HBV transmission modes [6], frequent and unprotected sexual intercourse, injection drug use (IDU), tattooing and other forms of skin piercing, and trauma [2,5,7]. The time period following prison release is also important in terms of increased risky behaviours (IDU and unsafe sex) [8]. Crofts et al. [9] showed that the annual incidence rate of HBV (per 100 person years) between two separate incarcerations was 12.6% (10 of 99 individuals seroconverted at subsequent entry). Such findings highlight the potential benefit of addressing hepatitis B prevention during imprisonment [9].

Prison settings provide a unique opportunity to implement HBV infection control measures for incarcerated individuals, thus reducing transmission within and outside the prison population [5]. However, given the epidemiological variability of HBV in European prisons, HBV prevention, and transmission control should be tailored to the local carceral context. This study aims to determine the prevalence of HBV serological markers, their associated risk factors, infection awareness and knowledge about modes of transmission/protection among inmates of the Champ-Dollon prison in Geneva, the largest pre-trial detention center in Switzerland.

## METHODS

### Study population and data extraction

A cross-sectional study was conducted at the Champ-Dollon pre-trial prison, at two distinct time points (2009 and 2011). During two varicella outbreaks, we conveniently offered to consenting participants of entire affected prison units a blood test and structured socio-demographic survey. There were no exclusion criteria. All questions were administered by a physician. The structured questionnaire included socio-demographic data, drug use, and sexual behaviour history (Table S1 in **Online Supplementary Document(Online Supplementary Document)**). Participants recruited in 2009 also agreed to answer a previously validated questionnaire about hepatitis B transmission and protection modes [6]. Consent forms and questionnaires were developed in nine languages (English, French, German, Italian, Spanish, Albanian, Russian, Chinese, and Greek). All data were anonymized. Parts of the survey were submitted three to five days after the blood test. During this period, seven participants were released and had therefore some missing data. Of note, there was no HBV-focused intervention between 2009 and 2011 that could have influenced the results. The Clinical Ethics Research Committee of the University Hospitals of Geneva (HUG) approved the study (EC: 09-137).

### Laboratory and data analysis

Every participant underwent serological testing for hepatitis B virus surface antigen (HBsAg), antibodies against HBV antigen (anti-HBc) and antibodies against surface antigen (anti-HBs) (EIA Architect system, Unilabs laboratory, Geneva). HBsAg positivity signalled chronic infection. The presence of anti-HBc antibodies and negative HBsAg indicated resolved/prior infection. The presence of anti-HBs (>10UI/l) in the absence of HBsAg and anti-HBc indicated vaccine-acquired immunity, even if occult HBV infections could not be excluded. These cases are rare [10] and were not considered as a specific HBV status in our study.

Continuous variables were summarized as mean and standard deviation, and categorical variables as absolute and relative frequencies. We calculated 95% confidence intervals of prevalence ratios [95% confidence interval CI] using Wilson's score interval. We also estimated how many people living in the canton of Geneva and having chronic HBV are susceptible to go through the correctional system. For this purpose, we used data on the number of adults living in the canton of Geneva (381 000 adults aged more than 18 years), the number of people being incarcerated each year in Geneva (1789 adults), the prevalence rate of HBV in the Swiss population (0.18%), and the prevalence rate of HBV in the Champ-Dollon prison estimated in this study [11-13].

Differences between groups were then tested using the χ^2^ test. The Fisher exact test was used when the expected value in 2x2 contingency tables was less than 5. Association between HBV infection (chronic or resolved) and other categorical variables (age, gender, region of origin) was evaluated in bivariate analysis. Participants with missing data were not included in the related analyses. Region of origin encompassed six regions of the world for participants’ home countries: Western Europe, Eastern Europe and the Balkans, North Africa, Sub-Saharan Africa, Latin America, and Asia. We stratified the study’s serological results based on known country-level HBsAg prevalence (HBV endemicity), using the WHO classification and recent publications: “low” (<2.00%), “lower-intermediate” (2.00-4.99%), “higher-intermediate” (5.00-7.99%), and “high” (≥8.00%) (10-13). A logistic regression was performed to identify factors associated with HBV infection. All variables with p-value <0.20 in the bivariate analyses were included in a multivariate model. Since Champ-Dollon is a pre-trial prison with a high turnover of detainees, people incarcerated in 2009 were not the same as those incarcerated in 2011. All analyses were performed for the whole sample (2009 and 2011) and then separately for 2009 and 2011 to test whether there were significant differences between the two time points. The statistically significant level was set to *P* < 0.05. All analyses were performed using SPSS for Windows (version 17.0) (IBM, Armonk, NY, USA) or OpenEpi (version 2.3.1) (open source software).

## Results

### Socio-demographic characteristics and sexual health risks

**Table 1** and **Table 2** summarize the socio-demographic and sexual behaviour risks among the participants: out of 281 inmates, 273 agreed to participate in the study (116 in 2009, 157 in 2011, participation rate: 97.2%).

**Table 1:** Socio-demographic characteristics among inmates at the Champ Dollon prison in Geneva, Switzerland, 2009 & 2011 (n = 273)

Characteristics	Total	2009	2011	*P*-value*
Sex (%, n):				
Male	100 (273)	100 (116)	100 (157)	–
**Region of origin (%, n):**				
Central and Eastern Europe	38.1 (104)	36.2 (42)	39.5 (62)	<0.001
Sub-saharan Africa	28.2 (77)	31.9 (37)	25.5 (40)	<0.001
North Africa	14.3 (39)	21.6 (25)	8.9 (14)	<0.001
Latin America	9.5 (26)	2.5 (3)	14.7 (23)	<0.001
Western Europe	29.2 (5)	6.9 (8)	10.8 (17)	<0.001
Asia	0.7 (2)	0.9 (1)	0.6 (1)	<0.001
Age (mean years, SD)	29.8 (9.0)	27.7 (7.2)	30.9 (8.8)	0.002
**Self-evaluated socioeconomic status (n, %):**				
Low	16.2 (43)	10.6 (12)	20.4 (31)	0.188
Moderate	77.4 (205)	83.2 (94)	73.0 (111)	0.188
High	6.4 (17)	6.2 (7)	6.6 (10)	0.188
**Level of education (n, %):**				
No school	3.0 (8)	4.5 (5)	2.0 (3)	0.126
At least some primary school	14.0 (37)	15.9 (18)	12.5 (19)	0.126
Finished primary school	10.9 (29)	10.6 (12)	11.2 (17)	0.126
At least some secondary school	21.6 (57)	15.9 (18)	25.7 (39)	0.126
Finished secondary school	40.3 (107)	46.9 (53)	35.5 (54)	0.126
Some university education	10.2 (27)	6.2 (7)	13.1 (20)	0.126
**Type of living area in childhood (n, %):†**				
Rural	19.8 (23)	19.8 (23)	–	–
Urban	80.2 (93)	80.2 (93)	–	–
**Legal status (n, %):†**				
Undocumented	63.7 (76)	63.7 (76)	–	–
Non-European migrant with residence permit in Switzerland	22.1 (25)	22.1 (25)	–	–
Swiss or European passport	10.6 (12)	10.6 (12)	–	–

SD – standard deviation

*χ^2^ tests or Fisher exact tests for categorical outcomes and *t* tests for continuous outcomes.

†Only asked to 113 out of 116 participants in 2009.

**Table 2:** Sexual health characteristics among inmates at the Champ-Dollon prison in Geneva, Switzerland, 2009 & 2011 (n = 273)

Characteristics (%, n)	Total	2009	2011	*P*-value*
**Sexual orientation:**				
Homosexual and/or bisexual	2.3 (6)	2.7 (3)	2.0 (3)	.807
Heterosexual	97.7 (258)	97.3 (109)	98.0 (149)	.807
**Number of sexual partners over lifetime:**				
0	0.4 (1)	0.9 (1)	0 (0)	.132
1	5.3 (14)	1.8 (2)	8.0 (12)	.132
2 to 5	21.3 (56)	21.2 (24)	21.3 (32)	.132
6 or more	73.0 (192)	76.1 (86)	70.7 (106)	.132
**Number of sexual partners in the 6 months before incarceration:**				
0	11.4 (30)	15.0 (17)	8.6 (13)	.465
1	42.7 (113)	39.8 (45)	45.0 (68)	.465
2 to 5	32.6 (86)	33.6 (38)	31.8 (48)	.465
6 or more	13.3 (35)	11.6 (13)	14.6 (22)	.465
**Age of first sexual intercourse (years):**				
≤16	53.2 (133)	40.5 (45)	48.9 (68)	.116
>16	46.8 (117)	59.5 (66)	51.1 (71)	.116
**Sexual intercourse with sex worker:**				
No	47.2 (125)	52.2 (59)	43.4 (66)	.349
Yes	52.8 (140)	47.8 (54)	56.6 (86)	.349
**Use of condoms during sexual intercourse:**				
Always	33.0 (87)	21.4 (24)	41.4 (63)	<.001
Sometimes	56.8 (150)	72.3 (81)	45.4 (69)	<.001
Never	10.2 (27)	6.3 (7)	13.2 (20)	<.001

*χ^2^ tests or Fisher exact tests.

Two thirds of the participants (63.7%) were undocumented (no Swiss or European Union (EU) passport or residence permit in Switzerland) (Table 1), and 38.1% originated from Central and Eastern Europe, 28.2% from sub-Saharan Africa, 14.3% from North Africa, 9.5% from America Latin, 9.1% from Western Europe, and 0.7% from Asia. Mean age was 29.8 years (SD 9.0). Half of the participants (50.7%) had completed high school. In terms of sexual health, 2.3% reported to be homosexual and/or bisexual. More than half (53.0%) of the respondents reported having had sexual activities with sex workers and having experienced their first sexual encounter before the age of 16. Two-thirds (67.0%) reported occasional or no condom use. The only significant differences between 2009 and 2011 were age (older participants in 2011, *P* = 0.002), region of origin (in 2011, there were fewer participants from North Africa and more from Latin America, *P* < 0.001), and use of condom (more participants reported “sometimes” in 2009, *P* < 0.001).

### Hepatitis B seroprevalence and region of origin

**Figure 1:**
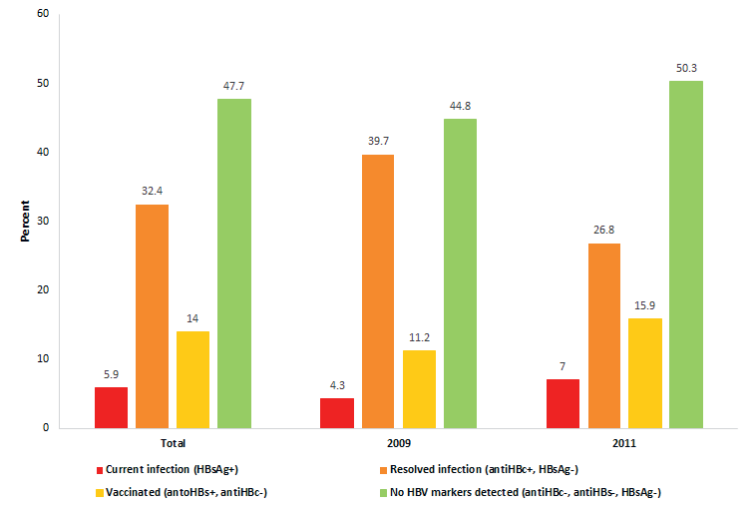
Hepatitis B markers among inmates at Champ-Dollon prison in Geneva, Switzerland, 2009 and 2011 (n = 273).

**Figure 1** summarizes the HBV serological profile of the study population: 5.9% (95%CI = 3.6-9.3) had a chronic infection (HBsAg+), 32.4% (95% CI = 32.7-44.1) had resolved HBV (anti-HBc+, but HBsAg-), and 14.0% (95% CI = 10.3-18.6) had a serological profile consistent with immunization (anti-HBc-, anti-HBs+). Just less than half of the inmates (47.7%, 95% CI = 41.9-53.7) had no detectable HBV markers, meaning that they were neither HBsAg carriers nor immune, and therefore susceptible to HBV infection. There was no significant difference between HBV status in 2009 and 2011 (*P* = 0.131 for the χ^2^ test). With a population of 381 000 adults in Geneva and a prevalence rate of 0.18% of HBV in the Swiss population, we can estimate that 686 persons per year are infected in the general population (381 000 × 0.0018). With 1789 adults incarcerated each year and a prevalence rate of 5.9% in Champ-Dollon prison, we can estimate that 106 persons are infected in the incarcerated population (1789 × 0.059). Therefore, 15.5% (106 out of 686 persons) of the infected persons go through Geneva’s prison for a given year.

In terms of anti-HBc positivity (ever infected with HBV), 72.2% (95%IC: 61.9-81.4) from sub-Saharan Africa were positive, 34.6% (95%CI = 26.2-44.2) from Central and Eastern Europe, and 13.2% (95% CI =  7.7-21.6) from other regions (primarily Western Europe, Latin America, and North Africa) (*P* < 0.001) (**Table 3**). The anti-HBc prevalence was statistically significant when aggregated by countries considered as “low” 9.1% (6/66), “lower-intermediate” 22.7% (17/75), “higher-intermediate” 46.3% (25/54), and “high” 72.7% (56/77), (*P* < 0.001). Among the eastern European block, 49.0% of detainees from the “high-intermediate” countries of Romania and Albania were positive for anti-HBc, compared to only 21.8% of those from the other Eastern European and Balkan countries (*P* = 0.004). Sub-Saharan African region was associated with presence of HbsAg (chronic HBV infection) with a prevalence of 15.6% vs 2% participants from other regions, all combined (*P* < 0.001). HBsAg endemicity per country also followed a statistically significant difference (*P* = 0.001): “low to lower-intermediate countries” 1.4% (2/141), vs “higher-intermediate to high countries” 10.7% (14/131).

**Table 3:** Association between positive HBV surface antigen (HBsAg), positive core antibody (AntiHBc) and sociodemographic and sexual health characteristics among inmates at the Champ-Dollon prison in Geneva, Switzerland, 2009 & 2011 (n = 273)

	n	HBsAg+/total (%)	*P*-value	AntiHBc+/total (%)*	*P*-value
**Region of origin:**					
Sub-Saharan Africa	77	12 (15.6%)	**<0.001†**	56 (72.7%)	**<0.001**
Other regions	196	4 (2.0%)		48 (24.6%)*	
**Age (years):**					
≥28	141	7 (5.0%)	0.51	54 (38.6%)*	0.91
<28	132	9 (6.8%)		50 (37.9%)	
**Education level:**					
Secondary school	191	8 (4.2%)	0.56†	70 (40.9%)*	0.72
Never to secondary school	74	5 (6.8%)		29 (39.2%)	
**Condom use for sexual protection:**					
Sometimes or never	177	9 (5.1%)	0.73†	70 (39.5%)*	0.41
Always use condoms	87	6 (6.9%)		30 (34.5%)	0.41
**Man who has had sex with men:**					
Yes	6	1 (16.7%)	0.60†	4 (66.7%)	0.30†
No	258	14 (5.4%)		96 (37.2%)*	
**Number of sexual partners (lifetime):**					
6 or more	192	8 (4.2%)	0.29	70 (36.5%)*	0.84
0 to 5	71	6 (8.5%)		27 (38.0%)	
**Number of sexual partners (6 months before incarceration):**					
6 or more	35	2 (5.7%)	0.99†	9 (25.7%)	0.12
0 to 5	229	13 (5.7%)		90 (39.3%)*	
**Age of first sexual intercourse (years):**					
≤16	133	6 (4.5%)	0.60	53 (39.8%)	0.25
>16	117	7 (6.0%)		38 (32.7%)*	
**Sexual intercourse with sex workers:**					
Never	125	9 (7.2%)	0.31	44 (35.2%)	0.39
At least once	140	6 (4.3%)		56 (40.3%)*	
**Injection drug use:**					
Yes	16	0 (0%)	0.78†	7 (43.8%)	0.65
No	253	15 (5.9%)		96 (38.0%)*	
**Type of living area:‡**					
Rural	23	0 (0%)	0.65†	14 (60.9%)	0.07
Urban	93	5 (5.4%)		37 (39.8%)	

*Not enough serum for antiHbc for one participant.

†Fischer exact test.

‡Only asked to 113 out of 116 participants in 2009.

### Hepatitis B seroprevalence and other factors

In bivariate analysis (**Table 3**), the results showed no association between HBV infection (chronic or resolved) and age, education level, number of sexual partners, age of first sexual activity, history of sexual activity with sex workers, same-sex sexual activities, injection drug use, rural or urban living, or “non-use” of condoms. In further sensitivity tests including all factors with *P*-values <0.20 (multivariate regression checks) only region of origin was significantly associated with presence of anti-HBc. Disaggregated analyses for 2009 and 2011 yielded similar results (not shown in **Table 3**).

### Knowledge about infection and vaccination history

Out of the 16 patients with chronic hepatitis B, 2 (12.5%) knew about their status. The first 116 participants who joined the study also answered questions about vaccination history: 15.0% were certain about prior vaccination, of whom 35.3% (5.3% of all participants) reported reception of 3 doses. Eight had anti-HBs suggestive of vaccination, 7 had resolved HBV (anti-HBc without HBsAg) and 2 were susceptible to HBV. Among the 30 detainees who reported no vaccination, 46.7% were immune post hepatitis B infection (anti-HBc+), but 10.0% had chronic hepatitis B (HBsAg+).

### Knowledge about transmission of and protection against HBV

Among the first 116 participants included in the study in 2009, 113 answered a questionnaire assessing their knowledge of and protection against HBV transmission (**Table 4**). Only 27% of participants knew that HBV could be transmitted by syringe exchange, 21% by razors or toothbrushes exchange, and 19% through tattoos. Overall 21% and 27% stated that using condoms and sterile syringes, respectively, could prevent HBV infection.

**Table 4:** Knowledge about hepatitis B transmission and prevention modes among inmates at the Champ-Dollon prison in Geneva, Switzerland, 2009 (n = 113)

Questions	% (n)
**Can you get hepatitis B from having unprotected sex?**	
Yes	24.8 (28)
No or I don’t know	75.2 (85)
**Can you get hepatitis B from sharing needles?**	
Yes	26.6 (30)
No or I don’t know	73.4 (83)
**Can you get hepatitis B from sharing toothbrushes or razors?**	
Yes	21.2 (24)
No or I don’t know	78.8 (89)
**Can one get hepatitis B by eating contaminated food?**	
No	9.7 (10)
Yes or I don’t know	90.3 (103)
**Can you get hepatitis B from being tattooed?**	
Yes	19.5 (22)
No or I don’t know	80.5 (91)
**Can a baby be infected with hepatitis B at birth, when born to a hepatitis B positive mother?**	
Yes	15.9 (18)
No or I don’t know	84.1 (95)
**Can drinking alcohol make liver disease worse if you have hepatitis B?**	
Yes	15.0 (17)
No or I don’t know	85.0 (96)
**Can one avoid hepatitis B infection by using condoms during sexual intercourse?**	
Yes	21.2 (24)
No or I don’t know	78.8 (89)
**Can one avoid hepatitis B infection by using sterile needles?**	
Yes	27.4 (31)
No or I don’t know	72.6 (82)

## DISCUSSION

### Hepatitis B seroprevalence

With regard to chronic hepatitis B, 5.9% of the surveyed population in Champ-Dollon were HBsAg carriers, which is 33 times higher than the proportion in the general Swiss population [14]. This prevalence ranks among the highest values recorded in prisons in Europe and in the United States [4,5,15]. Additionally, a simple estimation suggested that 15.5% of HBV infected people living in the Canton of Geneva go through the correctional system each year. Because these persons are among the most hard-to-reach in the community, the detention system has a civil responsibility facing the challenge of controlling these diseases. This is also a unique opportunity to treat this disease and protect other people of being infecting.

### HBV infection and associated risk factors.

The differences in HBV seroprevalence by region of origin found in the study are similar to the differences found in the literature, even by pre-determined stratification classes. Countries in sub-Saharan Africa are characterized by high percentages of HBsAg positivity (greater than 8%), which is reflected by the high prevalence found among migrants from these countries in Europe [16]. North African countries are classified at the “low” or “lower-intermediate” levels. In Western European countries, the prevalence is low. The epidemiology of hepatitis B in Eastern Europe (including Balkan countries) is more heterogeneous: Serbia, Croatia, Bosnia and Herzegovina, and Slovenia report low prevalence, while Albania and Romania have “high-intermediate” HBV endemicity [14,16-19]. About half (48%) of the study population and 88% of chronic hepatitis B cases came from countries with “higher-intermediate” (Albania, Romania) or “high” (sub-Saharan Africa) endemicity levels of HBsAg. These concordant findings show that prevalence figures among the general population in the country of origin can be extrapolated to prison populations in order to estimate risk of HBV by region of origin.

Sexual health risk factors (number of sexual partners, reported age of first sexual intercourse, history of activity with sex workers, and “non-use” of condoms) were not significantly related to infection in our study. These factors are known to be associated with HBV transmission [20-22]. It is possible that the sensitive nature of these questions led to an under-reporting of these sexual behaviours by prisoners. This however underscores that in prison populations, asking these sensitive questions may not always be useful in selecting subpopulations that are most at risk for HBV, given that many patients will not answer the question accurately.

This study did not show a statistically significant correlation between low education and chronic hepatitis B, contrariwise to previous studies, which demonstrated a clear link between low education status and HBV infection [20,23,24].

The relationship between age and hepatitis B is less clear in the literature: among some prisons in Europe, Australia and the United States, markers of HBV infection are higher in older age groups [25-27]. However, among prisons in sub-Saharan Africa (Nigeria, Ghana), younger age is associated with HBV infection [28,29]. We did not find evidence for this association in our sample. Further data are needed on this topic.

### HBV infection and knowledge about HBV status

Knowledge of having been infected by HBV among seropositive inmates was very low. Among 16 inmates with chronic HBV infection, only 2 (12.5%) knew that they were infected, which corresponds to values reported in the general Swiss population [30]. A previous study of medical files in this population (no systematic serological screening) showed a 1% prevalence of self-reported chronic HBV [31]. This underestimate corroborates that the majority of people living with chronic HBV infection in this population are unaware of (or ignore) their status- thus re-affirming the necessity for active HBV surveillance in prison. Furthermore, among the inmates reporting prior vaccination, 29% had antibody levels that would not ensure long-term protection (anti-HBs <100); 12% were HBV susceptible, and 41% possessed markers of past/resolved HBV infection. These results suggest that in the absence of serological markers, a verbal history about vaccination and immunity is not reliable in this population.

### Implications for HBV screening, vaccination strategies, and education

HBV serological screening has two objectives: 1) to diagnose chronic HBV infection, and 2) to detect people who are not appropriately immunized and need vaccination. Studies that incorporate cost-effectiveness measures recommend routine screening for chronic HBV (HBsAg positive) when the pre-test probability in the population is higher than 2% and when access to treatment for the majority of diagnosed patients is ensured (58-100% depending on the models) [32-34]. Access and adherence to the long antiviral treatment is challenging in the prison setting due to poor continuity of care and follow up, and risks of breakthrough resistance secondary to lapses in treatment [35]. Despite issues with treatment initiation, screening for HBV remains beneficial in the prison setting, as those diagnosed by routine screening could benefit from health education in order to 1) limit the risk of a downward evolution of the disease (for example, only 15% of our participants knew that alcohol consumption could worsen liver damage in the presence of HBV infection), and 2) modify unsafe behaviours that could increase the risk of further HBV transmission in prison and after release [36].

Given the prevalence of HBV in our study population, HBV prevention and control measures should be offered to inmates during their time in prison. This could benefit their health and also protect the community as a whole. For instance, Pisu, Meltzer, & Lyerla [37] demonstrated that the health care system benefits from allocating resources for prison HBV vaccination programs, and the United States Center for Disease Control recommends routine HBV immunization, chronic HBV screening, and HBV preventive health education while in prison [3].

Because HBV infection and the region of origin are significantly associated in this population, it could be proposed that patients from “high-intermediate” and “high” level HBV countries (who actually make up about half of the prison population: Albania, Georgia, Bulgaria, and countries in Sub-Saharan Africa), receive systematic screening for resolved and chronic HBV (anti-HBc and HBsAg). Non-immune individuals should receive immunization, while chronic HBV patients would benefit from intensive educational and therapeutic programs. Those from countries with “low” or “lower-intermediate” HBV levels would be vaccinated without testing.

An American study in 2002 using a similar questionnaire showed that 75% of their prison participants were aware of the primary modes of HBV transmission (considered less than ideal) [6]. Our results indicate an even lower level of HBV knowledge, thus reinforcing the potential impact of educational programs. Weinbaum et al. [3] have demonstrated that educational programs that target HBV prevention in prison are effective.

Finally, stigmatization acts as a barrier for prevention and treatment. Indeed, it often prevents people from seeking screening and treatment. Stigmatization is largely due to ignorance about the disease. Therefore, the prison environment is a unique opportunity to propose adequate education, prevention, and treatment for people at risk of being infected [38]. Since migrants are likely to be discriminated against, efforts should be made to avoid further stigmatization due to this infectious disease.

### Limitations and strengths

A limitation in this project is the convenience sample of the study. Nevertheless, this sample was representative of the prison population; recruitment bias was minor with a high participation rate (97%), and no policy of attribution of cells according to the origin of the prisoners (or any other criterion) existed. The study population represented respectively one-fifth and one-third of all prisoners incarcerated at the time of recruitment in 2009 and 2011. Because participants were asked to answer questions about themselves in terms of education (self-assessment) and sexual health (taboo questions), this information may have been less reliable due to reporting bias and social desirability, given that the questionnaire was completed face-to-face with a physician. Among participants with a serological pattern compatible with a resolved HBV (anti-Hbc pos/HbsAg neg), occult HBV infections were not excluded. Since these cases are rare, under-evaluation of chronic HBV prevalence in the study population would be very limited [10]. Future studies could focus on occult HBV infection among inmates. It would give an idea of the number of these cases, even if it concerns a very small proportion of active HBV cases. In this population, data about vaccination was confusing and participants did not reliably report their vaccination history. In terms of study strengths, we highlight the participation rate of 97%; these results are useful in the implementation of hepatitis B preventive measures in a population that lacks epidemiological data. It must be noted that this study population was entirely male and focused on a single prison in Switzerland. However, the study population is similar to that of other pre-trial Swiss prisons, which are also characterized by a high proportion of immigrants (81.4%) and men (94%) [39]. Therefore, these results are of importance to prison health providers in Switzerland, and also in all countries where the proportion of migrants in prison is high. In Europe, immigrants represent a high proportion of the correctional population. In 2012, foreigners accounted for 21% of the estimated 1.73 million people detained in the prisons of the 47 Member States of the Council of Europe. The vast majority of foreign detainees originated from low-income countries [40]. Therefore, an inclusive approach to global health needs to incorporate prison population, as incarcerated people have a disproportionate burden of HBV infection [41]. In addition to the risk of transmission in custody between inmates and to the staff, on release there is the risk of HBV being transmitted to the wider community, as infected inmates represent a risk for their partners at the return to freedom. Our results recall that origin must also be taken into account within the community among migrant populations native from HBV endemic countries.

## CONCLUSIONS

The prevalence of chronic HBV infection was 5.9% at the Champ-Dollon prison, 33 times higher than the general Swiss population [14]. The main risk factor for infection was region of origin. Other known socio-demographic risk factors for HBV were not reliable in our prison setting, thus limiting their use in risk-stratifying patients for hepatitis B. A targeted testing and vaccination approach based on the prisoner’s region of origin is a potential control strategy, especially because an important proportion of the infected and hard-to-reach population go through the detention system. Patient information, education, and counselling on HBV transmission modes should be offered to all inmates. An inclusive approach to global health needs to incorporate prison population, as incarcerated people have a disproportionate burden of HBV infection.
