# Supplementary material for: Hepatitis B prevalence, risk factors, infection awareness and disease knowledge among inmates: a cross-sectional study in Switzerland’s largest pre-trial prison
Source: J Glob Health. 2018 Jul 30;8(2):020407. doi: 10.7189/jogh-08-020407 (PMC6076584; doi:10.7189/jogh-08-020407)
Supplement: Online Supplementary Document [file jogh-08-020407-s001.pdf]

Online Supplementary Document

Getaz et al. Hepatitis B prevalence, risk factors, infection awareness and disease knowledge among inmates: a cross-sectional study in Switzerland's largest pre-trial prison

J Glob Health 2018;8:020407

S1 Table: Questionnaire items and missing data, HBV study, Champ-Dollon prison.

ITEMS	Missing data (n)	Reasons
1. Age	0	
2. HBsAg serology	0	
3. Anti-Hbc serology	1	Not enough serum
4. Anti-HBs serology	0	
5. Country of origin	0	
6. Region of origin	0	
7. Arrived in Western Europe (Years)	7	3 Not registrated<br>4 don't respond
8. Number of sexual partners over lifetime	10	7 released<br>3 don't respond
9. Number of sexual partners in the 6 months before incarceration	9	7 released<br>2 don't respond
10. Age of first sexual intercourse	22	7 released<br>10 don't respond<br>5 don't know
11. Sexual orientation	8	6 released<br>2 don't respond
12. History of sexual intercourse with sex workers	8	7 released<br>2 don't respond
13. Use of condoms during sexual intercourse	8	7 released<br>1 don't respond
14. Self-evaluated socioeconomic status	8	7 released<br>1 don't respond
15. Level of education	8	7 released<br>1 don't respond
16. History of intravenous drug use	4	4 released
17. Knowledge of HBV infection	5	4 released<br>1 don't respond
18. Type of living area in childhood*	0	0
19. Legal status*	3	3 released
20. Can one get hepatitis B from having unprotected sex?*		
21. Can one get hepatitis from sharing needles?*		
22. Can one get hepatitis B from sharing toothbrushes or razors?*		
23. Can one get Hepatitis B by eating contaminated food?*		
24. Can one get hepatitis B from being tattooed?*		

25. Can a baby be infected with Hepatitis B at birth, when born to a Hepatitis B positive mother?*		
26. Can drinking alcohol make liver disease worse if you have hepatitis B?*		
27. Can one avoid Hepatitis B infection by using condoms during sexual intercourse?*		
28. Can one avoid Hepatitis B infection by using sterile needles?*		

**Not asked to participants recruited in 2011 (n=157)*
